# Bone Metastases in Patients with Leiomyosarcoma: A Retrospective Analysis of Survival and Surgical Management

**DOI:** 10.1155/2022/6806932

**Published:** 2022-05-06

**Authors:** Christa L. LiBrizzi, Ashish Vankara, Christian F. Meyer, Adam S. Levin, Carol D. Morris

**Affiliations:** ^1^Division of Orthopaedic Oncology, Department of Orthopaedic Surgery, The Johns Hopkins University School of Medicine, Baltimore, MD, USA; ^2^Department of Medical Oncology, The Johns Hopkins University School of Medicine, Baltimore, MD, USA

## Abstract

**Background:**

Leiomyosarcomas (LMS) are malignancies with smooth muscle differentiation. Metastasis to the bone is not uncommon. The literature on the clinical course and management of such metastases is limited. Our study describes the clinical course of LMS to the bone, including survival rates, prognostic factors, and surgical management.

**Methods:**

We retrospectively reviewed 396 LMS patients presenting at an academic center between 1995 and 2020. We included LMS patients diagnosed with bone metastases and excluded patients with primary LMS of bone. We evaluated survival time with the Kaplan–Meier survival method and used Cox's proportional hazards regression analysis to determine factors associated with survival.

**Results:**

Forty-five patients with LMS (11%) had bone metastases. The most common LMS subtypes with bone metastases were uterine (*N* = 18, 40%) and retroperitoneal (*N* = 15, 33%). Bone metastasis was not an independent predictor of mortality by Cox regression analysis (HR 1.0, 95% CI: 0.67–1.5). Patients more frequently metastasized to the axial (*N* = 29, 64%) than to the appendicular (*N* = 5, 11%) skeleton. Bone was the first site of metastasis in 13 patients (29%). Patients presented with bone metastases at a median of 32.7 months (IQR: 5.2, 62.6) after initial LMS diagnosis. Twelve patients (27%) sustained a pathologic fracture. Twenty (44%) required surgical management, with 30 surgeries total. Three (15%) had a failure of reconstructive constructs. The median overall survival time was 69.7 months (IQR: 43.2, 124.5). There were no associations between the LMS subtype and survival. Pathologic fracture was an independent predictor of mortality by Cox regression analysis (HR 5.4, 95% CI: 1.8–16).

**Conclusion:**

The majority of patients with metastatic LMS to bone survive greater than 5 years and frequently require surgical intervention. Extended survival in this patient population should inform fixation and implant choice. No anatomic subtype was associated with risk for bone metastases. Pathologic fracture was associated with worse survival.

## 1. Introduction

Leiomyosarcomas (LMSs) are heterogenous, malignant mesenchymal tumors that predominantly affect the soft tissue and organs of the abdomen, pelvis, and large blood vessels [1, 2]. The most common subtypes of LMS are uterine, soft tissue of the retroperitoneum, and soft tissue of the extremities [3]. While the prognosis and aggressiveness of LMS vary by subtype, oncologic management is largely similar in the form of wide surgical excision because the survival benefits of chemotherapy and radiation are unclear [2]. LMS carries a risk of metastatic spread, with the lung and liver being the predominant sites of metastasis [4]. Patients with LMS also have a risk for bone metastases [5].

The incidence of bone metastases in LMS is unknown. Further, the prognosis and treatment of LMS bone metastases are infrequently discussed, and the literature is limited to case reports and small case series that focus on spine metastases [6, 7]. The overall goal of treatment for patients with the metastatic bone disease is to reduce skeletal-related events, thus enabling reduced pain and increased quality of life. Improved survival after wide excisions of extrapulmonary metastases in patients with oligometastatic sarcomas, including leiomyosarcomas, has been demonstrated [8–11]. Surprisingly, there is no literature on the management of appendicular bone metastases from LMS. Therefore, in our study, we asked the following questions: how frequently does LMS metastasize to bone, and what is the clinical course of LMS bone metastases?

Our objective was to determine 1) correlations between the anatomic subtypes of LMS and the development of bone metastases; 2) the clinical characteristics of bone metastases in LMS; 3) the incidence and characterization of surgical management of bone metastases in LMS; 4) survival rates and associations with survival in patients with metastatic LMS to bone.

## 2. Materials and Methods

We received institutional review board approval for this study. We retrospectively reviewed all records of patients with biopsy-proven LMS who presented to our academic institution between 1995 and 2020 (*N* = 396, Table 1). Of the 396 patients, 273 were female (69%) and 123 were male (31%). Patients were most commonly Caucasian (66%) and African American (23%). There were fifteen subtypes of LMS, the most frequent being retroperitoneal (*N* = 125, 32%), uterine (*N* = 124, 31%), soft tissue of the extremity (*N* = 71, 18%), and dermal/cutaneous (*N* = 13, 3%). We grouped nonuterine gynecologic LMS subtypes (i.e., vaginal and vulvar) together as a gynecologic subtype and male reproductive LMS subtypes (e.g., testicular and seminal vesicle) together as a male reproductive subtype for analysis. We also grouped head, eyes, ears, nose, and throat (H&NH&N) LMS together for analysis (H&NH&NH&N subtype). Three patients (0.8%) presented with diffusely metastatic disease and anaplastic pathology, which resulted in the inability to determine the LMS subtype. Of the 396 patients, 94 were lost to follow-up at a mean follow-up time of 65 months. These patients were included for analysis.

For the final analysis, we included patients with LMS who had bone metastases. We excluded any patients without metastases to the bone (*N* = 344) or with primary LMS of bone (*N* = 7). Forty-five patients met the inclusion criteria.

We recorded patient age at initial diagnosis, sex, race, ethnicity, subtype of LMS, size of the tumor, histologic grade, presence of visceral metastases, presence of bone metastases, anatomic location of metastases, imaging characteristics of bone metastases, number of bone metastases, diagnostic imaging modalities, use of radiotherapy to treat bone metastases, prior chemotherapy, prior radiotherapy, and survival. In addition, we recorded pathologic fractures sustained before surgery, pathologic fractures sustained after nonoperative management, type of management (nonoperative versus operative), type of operative management, recurrence of LMS after operative management, postoperative complications, failure of operative management, and revision surgeries. We measured overall survival time from the date of initial diagnosis to time of death or last known date of follow-up. We measured implant survival time as time from initial surgery to time of death or last known date of follow-up, as long as there were no construct complications. We measured time to implant failure from the date of initial surgery to time of construct failure.

### 2.1. Statistical Analysis

We used Fisher's exact test to evaluate the association between LMS subtypes and bone metastases and the Kaplan–Meier survival method to evaluate survival time among patients with LMS metastases to bone. Differences in survival curves were compared with a log-rank test. We used multivariate Cox proportional hazards regression analysis to estimate hazard ratios for factors associated with survival. Statistical significance was considered at *p* < 0.05. All analyses were performed with Stata, version 17.0 (StataCorp LLC, College Station, TX).

## 3. Results

### 3.1. Patient Demographics

Of the 45 patients with metastatic LMS to the bone, 31 (69%) were female and 14 (31%) were male (Table 2). The mean age at initial LMS diagnosis was 56.2 ± 1.8 years old. The most common LMS subtypes with bone metastases were uterine (*N* = 18, 40%), retroperitoneal (*N* = 15, 33%), and extremity soft tissue (*N* = 8, 18%). The primary LMS tumor size was available in 34 patients: in 10 patients, it was ≤5 cm in size; in 13 patients, it was >5 cm but ≤10 cm; in nine patients, it was >10 cm but ≤15 cm; in two patients, it was >15 cm but ≤20 cm. Twenty-nine patients had high-grade primary LMS, whereas 15 had intermediate grade and one patient had low-grade primary LMS. Thirteen patients (29%) had bone as their first site of metastasis. Three patients had bone metastases only. Patients presented with bone metastases at a median of 32.7 months (IQR: 5.2, 62.6) after initial LMS diagnosis. All bone metastases were diagnosed by CT or plain radiographs. Thirteen patients (29%) underwent PET, and 14 (31%) underwent bone scanning for routine surveillance imaging of their sarcoma. Only three patients had both PET and bone scans. Most patients were determined to have high-grade histology on pathology review (*N* = 29, 64%). Forty-one patients (91%) had chemotherapy and 37 patients (82%) had radiotherapy to sites other than bone metastases before bone metastasis diagnosis. Forty-two patients (93%) also had evidence of visceral metastases. Patients presented with visceral metastases at a median of 20.6 months (IQR: 1.1, 48.5) after initial diagnosis. There were 32 patients who had died with or from disease and 12 who were still living with the disease. One patient was lost to follow-up out of the 45 patients with metastatic LMS to bone, with a total of 94 patients lost to follow-up from the entire LMS cohort.

### 3.2. Characterization of Bone Metastases

**Figure 1:**
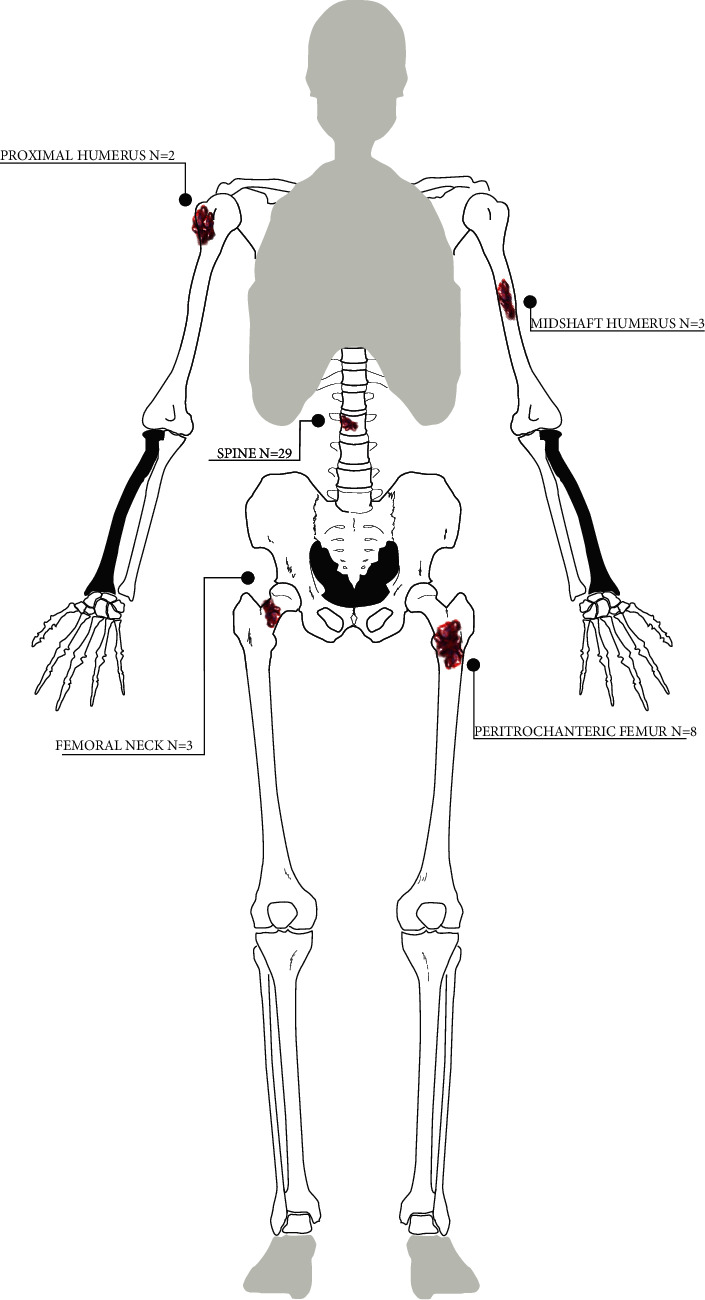
Anatomic locations of bone metastases in leiomyosarcoma patients.

There was no association between any LMS subtype and the development of bone metastases by Fisher's exact test (*p*=0.20). Patients with metastatic LMS to bone more frequently had metastases to the axial skeleton (*N* = 29, 64.4%; Table 3) than to the appendicular skeleton (*N* = 5, 11.1%). The thoracic spine (*N* = 22) and lumbar spine (*N* = 15) were common sites afflicted in the axial skeleton.  Figure 1 illustrates the anatomic location of appendicular metastases. Patients with spine metastases had lesions within the vertebral body (*N* = 17, 59%) or posterior elements (*N* = 12, 41%). The femur (*N* = 11) and the humerus (*N* = 5) were the most common sites of appendicular metastasis. Most bone metastases were lytic lesions (*N* = 32, 71%). Patients were more likely to have multiple bone metastases (*N* = 32, 71%) than an isolated bone metastasis (*N* = 13, 29%). In the 13 patients who underwent a PET scan, all lesions demonstrated fluorodeoxyglucose (FDG) avidity. Of the 14 patients who underwent a bone scan, 11 patients (79%) did not demonstrate technetium-99 uptake in the bone metastases. The three patients with both a PET scan and a bone scan all had FDG avidity with no avidity on the bone scan. Thirty-one patients (69%) underwent radiotherapy for the management of bone metastases. Twenty-nine of the 31 patients (94%) received palliative radiotherapy. Total radiation dose and fractionation schedule were not available for analysis. Seventeen patients (38%) received antiresorptive therapy. Twelve patients (27%) sustained a pathologic fracture due to metastatic disease.

### 3.3. Surgical Management of Bone Metastases

**Figure 2:**
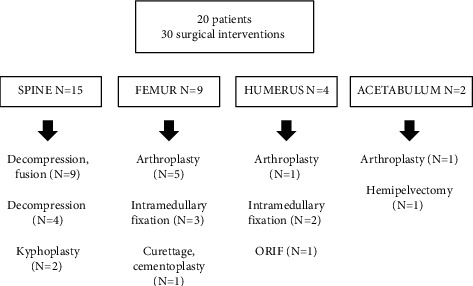
Surgical management of metastatic leiomyosarcoma to the bone.

There were 30 total surgical interventions for bone metastases in 20 patients (Figure 2). The remaining 25 patients did not require surgical intervention for their bone metastases. Five patients who did not require surgical intervention are alive with the disease. Six patients required two surgical interventions and three patients required three or more surgeries. Twelve patients (60%) underwent prophylactic fixation, whereas eight patients (40%) had an operative intervention for pathologic fracture fixation. The median time from bone metastasis diagnosis to surgical intervention was 2.3 months (IQR 0.6, 11.4).

The most common operative site was the spine (*N* = 15, 50%), followed by the femur (*N* = 9, 30%) and the humerus (*N* = 4, 13%). Nine patients underwent decompression and fusion of the spine, four underwent decompression alone, and two underwent kyphoplasty. Among the femur surgery patients, five underwent arthroplasty, three had intramedullary fixation, and one had curettage and cement augmentation. Among the humerus surgery patients, one had hemiarthroplasty of the shoulder, one underwent fixation with plate and screws, and two had intramedullary fixation. Two patients had acetabular bone metastases; one underwent hemipelvectomy and the other underwent curettage, acetabular reconstruction, and total hip arthroplasty. Among the patients who had hardware implanted, the median implant survival time was 14.8 months (IQR: 6.0, 32.6).

Six postoperative complications occurred in five patients. Three patients had a failure of implants requiring operative intervention. Time to implant failure was a median of 12.7 months (IQR: 12.5, 23.6). One patient had metastasis in their proximal femur that required intramedullary fixation for prophylactic stabilization, which was complicated by a thigh hematoma three weeks postoperatively that was irrigated and debrided. One patient with metastasis at L4 requiring L2-S4 posterior spinal fusion and decompression developed new pain five months postoperatively. The patient was found to have a recurrence around L4 but no spinal instability or thecal compression, and so they were treated with stereotactic radiotherapy.

Another patient presented with lumbar back pain and was found to have a metastatic disease with a pathologic compression fracture at L2, which was initially treated with vertebroplasty. Twelve months later, the patient had acute worsening of pain and there was radiographic evidence of recurrence and spinal instability that necessitated T11-L4 posterior spinal fusion, decompression, and L2 corpectomy.

One patient had metastasis to the subtrochanteric region of the femur treated prophylactically with intramedullary fixation. Twenty-three months later, they had pain while ambulating with radiographic evidence of recurrence around the lag screw. They were treated with curettage, ablation, and cement augmentation and were alive with the disease at 105 months. One patient had two postoperative complications at separate sites of bone metastases. First, the patient had a proximal femur metastasis that was initially treated with curettage, excision, and cement augmentation. Forty-eight months postoperatively, they had pain and progression of disease that required a proximal femur replacement (PFR). Second, sixteen months after PFR, they had progression of proximal humeral metastases, despite radiotherapy treatment, and underwent curettage and prophylactic plate stabilization. The patient presented ten months later with pain and radiographic evidence of loosening of hardware and fracture. They subsequently underwent removal of the hardware and intramedullary nail fixation. The patient survived for nearly 13 months postoperatively with no further complications.

### 3.4. Survival

**Figure 3:**
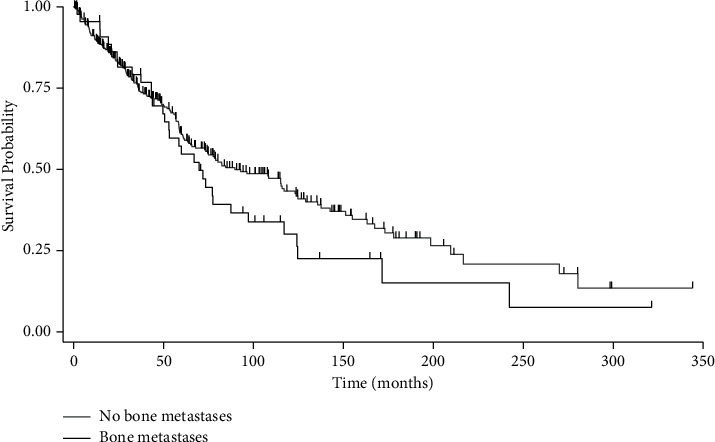
Kaplan–Meier survival curves by overall survival of patients with leiomyosarcoma without bone metastasis compared to those with bone metastases. Vertical hash marks represent censored patients.

The 2-year, 5-year, and 10-year survival rates of all 396 patients with leiomyosarcoma were 72%, 39%, and 16%. The 2-year, 5-year, and 10-year survival rates of patients with metastatic LMS to bone were 82%, 51%, and 18%, respectively. All patients with LMS, including those lost to follow-up, had a median survival time of 82.3 months (IQR: 36.4, 198.5). There was no significant difference in survival between patients with bone metastases and those without bone metastases by log-rank test (Figure 3, *p*=0.17). Bone metastases did not increase the risk of mortality on Cox regression analysis (Table 4, *p*=0.97). Primary LMS of the extremity soft tissue, dermal, and male reproductive system subtypes were independent predictors of survival with an associated decreased risk of mortality. An unknown primary LMS subtype diagnosis was an independent predictor of survival with an associated 7-fold increased risk of mortality by way of multivariate Cox regression analysis.

**Figure 4:**
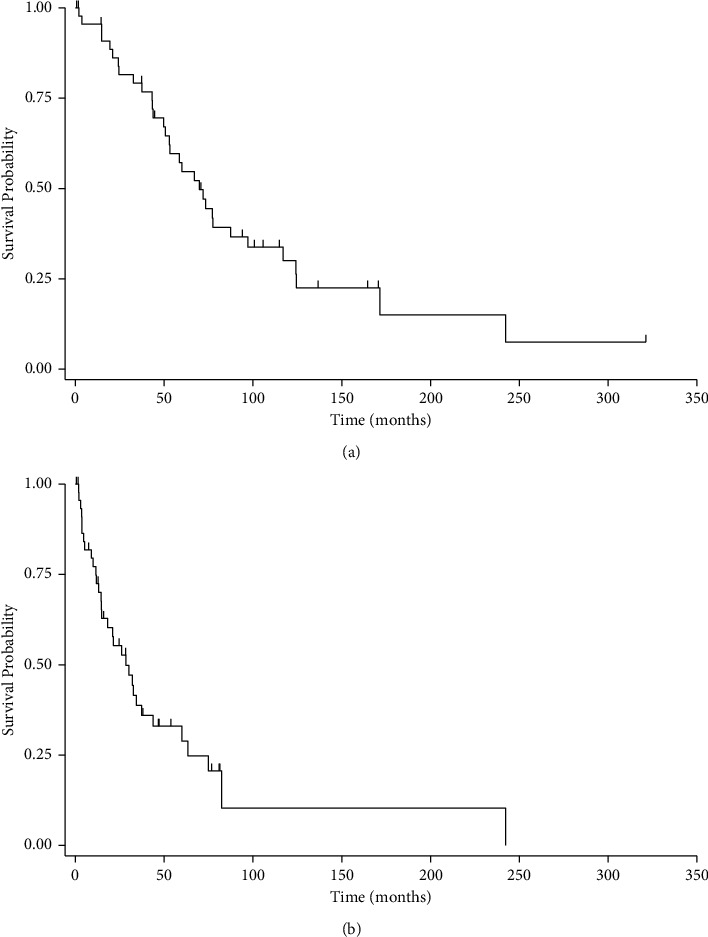
Kaplan–Meier survival curves by overall survival of all patients with metastatic leiomyosarcoma to bone from time of initial diagnosis (a) and survival after diagnosis of bone metastases (b). Vertical hash marks represent censored patients.

**Figure 5:**
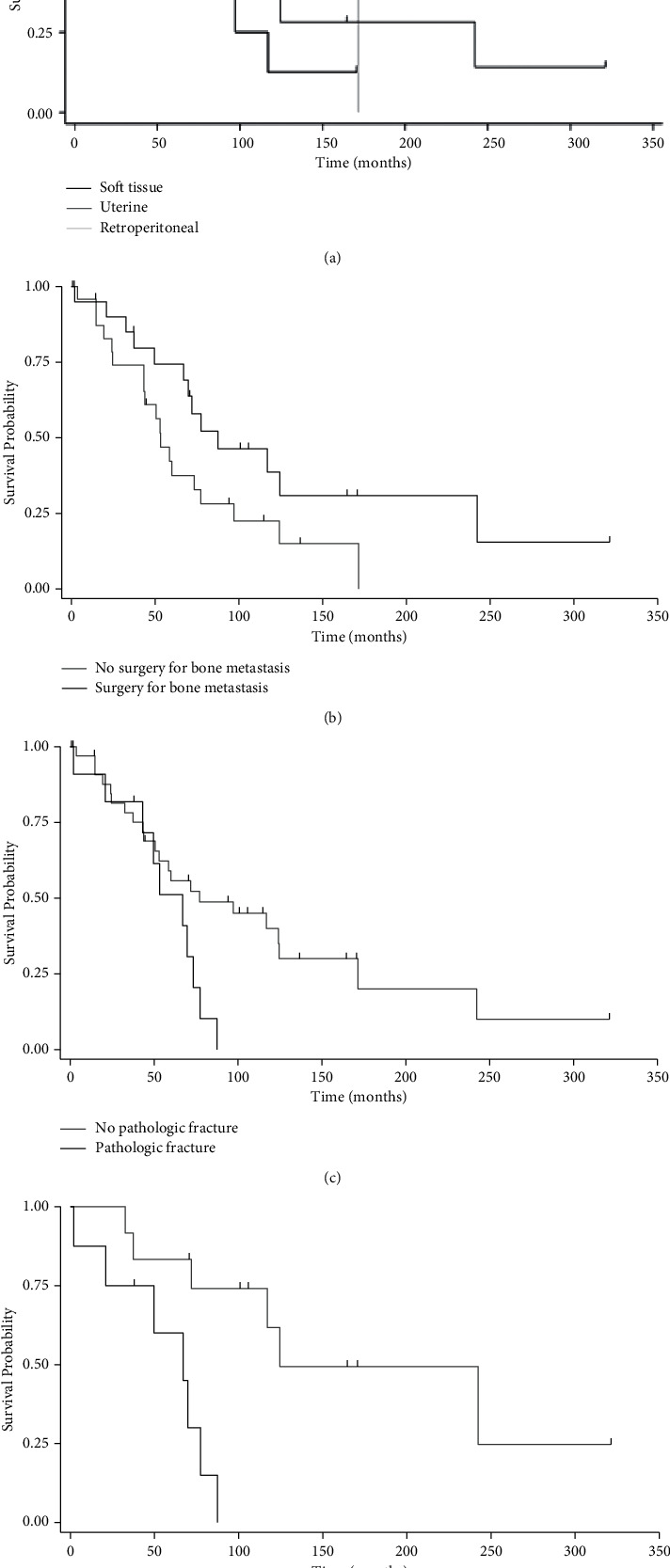
Kaplan–Meier survival analysis of patients with metastatic leiomyosarcoma according to subtypes of LMS, *p*=0.56 (a); surgical intervention for bone metastases, *p*=0.06; (b) pathologic fracture of bone metastases, *p*=0.04 (c); indication for fixation, *p*=0.002 (d). Vertical hash marks represent censored patients.

Patients with bone metastases had a median overall survival time of 69.7 months (IQR: 43.2, 124.5). In contrast, patients' median survival time after diagnosis of bone metastases was 28.4 months (IQR: 11.4, 63.3).  Figure 4 illustrates Kaplan–Meier survival curves by overall survival and survival from time of bone metastasis diagnosis. There were no associations between LMS subtype and survival (Figure 5). There was a positive association between survival and surgical intervention for bone metastases, although this did not reach statistical significance (*p*=0.06). There was a negative association between survival and surgical intervention for pathologic fracture when compared to prophylactic fixation (*p*=0.002). Pathologic fracture was an independent predictor of mortality, with an associated 5.4-fold increased risk of mortality through multivariate Cox regression analysis (Table 5, *p*=0.002).

## 4. Discussion

Leiomyosarcomas are one of the most common soft tissue sarcomas. They can present nearly anywhere within the body and have a highly variable clinical course [2]. While visceral metastases are more common, bone metastases are not infrequent and can lead to significant morbidity [12]. There is a gap in the literature regarding the presence of bone metastases in LMS and their subsequent clinical courses and management. We found that bone metastases were present in 11% of patients who had biopsy-proven LMS over 25 years at a single institution. No particular subtype of LMS had a greater predilection for bone metastases. While the spine was the most common site afflicted, the appendicular skeleton was also frequently affected. Patients more often had multiple bone metastases than the oligometastatic disease. Management of bone metastases often consisted of radiotherapy, but nearly 50% of patients required at least one surgery, and 20% needed revision surgeries some years postoperatively. Many patients with bone metastases had extended survival, as the median overall survival was 5 years, with pathologic fractures being an independent predictor of mortality. Our study demonstrates that bone metastases in LMS are fairly common and can be successfully managed with surgery. This study also suggests that prophylactic operative intervention may be important in extending survival.

Patients with LMS tend to have longer survival times than patients with other sarcomas, such as undifferentiated pleomorphic sarcomas or angiosarcomas, at an equivalent stage [13]. Gootee et al. [14] analyzed patients with LMS in the National Cancer Database for predictors of survival. In their study, the median survival time was 95.1 months with a 5-year survival rate of 59.8%. They found an increased risk of mortality with LMS with origins in the female reproductive system, stage IV disease at diagnosis, and macroscopic residual tumor after surgery. In our study, in patients with metastatic LMS to the bone, the median overall survival time was 60 months, with a median survival time of 21.3 months after diagnosis of bone metastases. Further, our 5-year survival rate was 51% in LMS with bone metastases, which is comparable to the Gootee et al. study, which included all patients with LMS. Our study did not demonstrate significant survival time differences in patients with bone metastases compared to those without. Further, bone metastases were not an independent predictor of mortality. While we did not observe differences in survival or propensity for bone metastases by particular LMS subtype, our sample size was relatively small compared to the above study. Our study demonstrates that patients with metastatic LMS to the bone have an extended overall survival time and survival time after diagnosis of bone metastases, a finding that could be used to guide treatment algorithms for future patient management.

There are overlapping indications for different surgical implants in the management of bone metastases. One area of frequent debate is the best choice of fixation methods for proximal femur metastases, particularly between endoprosthetic reconstruction (EPR) and intramedullary nail fixation (IMN). The literature has documented similar postoperative outcomes, but IMN has a notable increased risk of mechanical failure compared to EPR [15–17]. Hindiskere et al. [18] studied 70 patients with proximal femur metastases who underwent IMN (*N* = 37) compared to EPR (*N* = 33) and demonstrated no difference in intraoperative blood loss or surgical time. Patients treated with IMN had significantly greater rates of local recurrence and decreased local recurrence-free survival; ten patients required revision surgeries at a mean of 11.7 ± 14.7 months. Harvey et al. [19] also reported greater longevity and significantly reduced risk of mechanical failure with EPR compared to IMN in proximal femur metastases. In their study, IMN had an increased risk of mechanical failure with increasing patient survival. They noted 12 postoperative complications in the IMN group and six patients who were converted to EPR due to nonunion. Steensma et al. [17] compared surgical fixation failure rates between EPR, IMN, and open reduction and internal fixation (ORIF) in 298 patients with both impending and pathologic proximal femur fractures. EPR had a lower implant failure rate (3.1%) than IMN (6.1%) and ORIF (42.1%). In their study, EPR often failed due to dislocation, whereas IMN and ORIF failed due to disease progression or nonunion. In our study, three patients had four implant failures due to progression of the disease, requiring reoperation at a median of 12.7 months postoperatively. None of the failures involved endoprosthetic reconstruction and there were no prosthetic dislocations. While surgeons need to weigh the morbidity of surgical management, in patients with otherwise stable metastatic LMS, treatment along a primary bone sarcoma paradigm rather than IMN may be warranted in this patient population with extended survival.

Pathologic fractures are a feared skeletal-related event in metastatic bone disease, with significantly related literature reflecting efforts to accurately predict and prevent their occurrence [20–23]. Patients often present with debilitating pain, loss of function in the limb, and decreased quality of life, and they may require extended hospitalization. Previous literature has suggested that pathologic fracture may be associated with worse overall survival in both primary sarcomas and metastatic carcinomas. Our study found that pathologic fracture was an independent predictor of mortality and increased the risk of mortality by greater than 5-fold in patients with metastatic LMS. We also demonstrated positive associations with survival when metastatic LMS lesions were fixed prophylactically compared to fixation of pathologic fractures. In addition, we observed a trend of improved survival in LMS patients who underwent surgical management compared to those who underwent nonoperative management of bone metastases. While surgical indications favor a healthy patient population, these results suggest that prophylactic fixation in patients with metastatic LMS to bone improves survival and patients may benefit from early prophylactic fixation. Definitive correlations between survival and early surgical management of LMS bone metastases require a larger study cohort.

Surveillance imaging in patients with sarcoma can be highly variable in terms of the imaging modalities ordered and the time intervals for ordering them. Another layer of nuance is added when considering whether the surveillance is for local recurrence, pulmonary metastases, extrapulmonary metastases, or all of the above. Cipriano et al. [24] recently published a review on sarcoma surveillance emphasizing the variance due to provider preference and sarcoma diversity. Evidence for intervals and specific modalities for extrapulmonary metastases is particularly lacking. This is likely due to extrapulmonary metastases being relatively rare compared to pulmonary metastases and also being associated with a worse overall prognosis. However, in our study, we noted that many patients with LMS had extended overall survival time and survival time after diagnosis of bone metastases. This suggests a relatively indolent behavior of these metastases and patients have minimal complications after surgical management. In our cohort, most bone metastases were initially diagnosed on surveillance CT imaging, but nearly one-third of patients had supplementary imaging by PET or bone scanning. In our study, the majority of patients had PET-avid lesions but lacked uptake on bone scans. In the three patients with both PET and bone scans, all had PET-avid lesions with no evidence of uptake on bone scans. Previous studies also have found LMS bone metastases to be PET-avid [25]. Our findings suggest that patients with LMS may benefit from being grouped with sarcoma subtypes that commonly incorporate PET scans as part of surveillance imaging, based on both their extended survival and the incidence of bone metastases. This study also suggests that bone scanning may be an unreliable screening and surveillance method for identifying bone metastases in LMS.

Our study has several limitations. First, our data were derived from a single, tertiary cancer center. Thus, our patient population may not represent the entire LMS population. Second, because this was a retrospective study, we were limited to electronic medical record data, and it is possible that clinical events were not documented in the record or that they were incorrectly transcribed. Despite this limitation, the electronic medical record allows greater access to documentation of care, including outside of our institution, perhaps allowing for a more complete dataset. Additionally, with a retrospective design, we were unable to control for possible confounders. We did not quantify metastatic tumor burden in this study as most patients had diffuse visceral metastases and bone metastases due to lack of feasibility. However, we did assess systemic therapies, the number and location of bone metastases, and locations of visceral metastases as variables associated with survival. Further prospective studies analyzing the interactions between tumor burden, functional status, and systemic therapy would greatly improve our understanding and risk stratification. Due to loss to follow-up in patients without bone metastases after an average of 65 months, there may be a bias in the survival times and associations between patients with metastases and those without. Despite these limitations, this study collected comprehensive data over 25 years and described a spectrum of clinical courses in patients with metastatic LMS.

## 5. Conclusion

Patients with metastatic leiomyosarcoma to the bone have prolonged overall survival and survival time after bone metastasis diagnosis. Diagnosis is often made incidentally on surveillance imaging, and many patients eventually require surgical intervention. The surgeries and implants chosen should ideally outlive the patient's life expectancy to reduce the surgical burden. Further analysis is needed to determine which patients warrant early prophylactic fixation to reduce the risk of pain, loss of function, and worse survival following pathologic fracture.

## Figures and Tables

**Table 1:** Demographics of all 396 patients with biopsy-proven leiomyosarcoma.

Demographics	N (%)
Sex	
Female	273 (69%)
Male	123 (31%)
Race	
Caucasian	262 (66%)
African American	92 (23%)
Asian	20 (5.0%)
Hispanic	2 (1.0%)
Other	20 (5.0%)
LMS subtype	
Retroperitoneal	125 (32%)
Uterine	124 (31%)
Extremity soft tissue	71 (18%)
Dermal/subcutaneous	13 (3.3%)
Male reproductive	13 (3.3%)
Primary bone	7 (1.7%)
Peritoneal	7 (1.7%)
Gastrointestinal	7 (1.7%)
Inferior vena cava	6 (1.5%)
Gynecologic	6 (1.5%)
Liver	4 (1%)
H&NH&N	4 (1%)
Mediastinal	2 (0.5%)
Lung	2 (0.5%)
Breast	2 (0.5%)
Unknown	3 (0.75%)

**Table 2:** Demographics of the 45 patients with metastatic LMS to bone.

Demographics	N (%)
Sex	
Female	31 (69%)
Male	14 (31%)
Age (years)	
Race	
Caucasian	33 (73.3%)
African American	10 (22.2%)
Other	2 (4.4%)
LMS subtype	
Uterine	18 (40%)
Retroperitoneal	15 (33%)
Extremity soft tissue	8 (18%)
H&N	1 (2.2%)
Lung	1 (2.2%)
Unknown	2 (4.4%)
Visceral metastases	42 (93%)
Median survival time (months, IQR)	60 (37.2, 100)

**Table 3:** Characterization of bone metastases.

Variable	N (%)
Location	
Axial	29 (64.4%)
Appendicular	5 (11.1%)
Both	11 (24.4%)
Lesion characterization	
Lytic	32 (71%)
Sclerotic	4 (9%)
Mixed	10 (20%)
Number of bone metastases	
Single	13 (29%)
Multiple	32 (71%)
Radiotherapy to bone metastases	31 (69%)
Pathologic fracture of bone metastasis	12 (27%)
Surgical management of bone metastases	20 (45%)
Indication for fixation	
Prophylactic fixation	12 (60%)
Pathologic fracture fixation	8 (40%)

**Table 4:** Cox regression analysis of survival in 396 patients with leiomyosarcoma.

Variable	Hazard ratio (95% CI)	*P*
Presence of bone metastases	1.0 (0.67–1.5)	0.97
Primary LMS^†^		
Bone	0.35 (0.10–1.1)	0.08
Extremity soft tissue	0.46 (0.29–0.72)	0.001^*∗*^
Dermal	0.21 (0.06–0.66)	0.008^*∗*^
Uterine	0.93 (066–1.3)	0.70
Peritoneal	1.5 (0.55–4.2)	0.41
Lung	2.2 (0.55–9.2)	0.26
Breast	0.72 (0.10–5.2)	0.75
Gynecologic	0.29 (0.07–1.2)	0.08
Male reproductive	0.33 (0,12–0.91)	0.03^*∗*^
Gastrointestinal	0.84 (0.26–2.7)	0.77
Inferior vena cava	0.91 (0.28–2.9)	0.87
Liver	2.8 (1.0–7.8)	0.05
H&N	0.27 (0.04–2.0)	0.20
Mediastinal	0.63 (0.09–4.6)	0.65
Unknown	7.0 (2.1–23.6)	0.002^*∗*^

^†^Retroperitoneal; ^*∗*^statistically significant (*p* < 0.05).

**Table 5:** Cox regression analysis of survival in 45 patients with metastatic leiomyosarcoma to the bone.

Variable	Hazard ratio (95% CI)	*P*
Primary LMS^*∗*^		
Extremity soft tissue	2.5 (0.57–11)	0.23
Uterine	0.82 (0.27–2.5)	0.72
Lung	125 (5.3–2998)	0.003
H&N	0.59 (0.05–6.7)	0.67
Unknown	9.4 (1.7–52)	0.01
Location of bone metastasis^†^		
Appendicular	0.90 (0.11–7.6)	0.92
Axial	0.64 (0.22–1.8)	0.41
Sex^‡^	1.1 (0.26–4.8)	0.88
Visceral metastases	1.5 (0.27–8.1)	0.65
Number of metastases^||^	1.3 (0.33–5.5)	0.69
Pathologic fracture	5.4 (1.8–16)	0.002
Surgical management	0.41 (0.16–1.0)	0.06

^*∗*^Retroperitoneal; ^†^metastases in axial and appendicular locations; ^‡^female; ^||^one metastasis.

## Data Availability

This study uses data from patient records at the single institution; thus, raw data are not available for public release.
